# Hierarchical Fe_3_O_4_-reduced graphene oxide nanocomposite grown on NaCl crystals for triiodide reduction in dye-sensitized solar cells

**DOI:** 10.1038/s41598-018-38050-z

**Published:** 2019-02-06

**Authors:** Viyada Harnchana, Sujinda Chaiyachad, Samuk Pimanpang, Chatree Saiyasombat, Pornjuk Srepusharawoot, Vittaya Amornkitbamrung

**Affiliations:** 1 Department of Physics, Faculty of Science, Khon Kaen University, Khon Kaen, 40002 Thailand; 2 Institute of Nanomaterials Research and Innovation for Energy (IN-RIE), NANOTEC -KKU RNN on Nanomaterials Research and Innovation for Energy, Khon Kaen University, Khon Kaen, 40002 Thailand; 3 Intergrated Nanotechonology, Khon Kaen University, Khon Kaen, 40002 Thailand; 4 Thailand Center of Excellence in Physics, Commission on Higher Education, Bangkok, 10400 Thailand; 5 Department of Physics, Faculty of Science, Srinakharinwirot University, Bangkok, 10110 Thailand; 6 Synchrotron Light Research Institute (Public Organization), Nakhon Ratchasima, 30000 Thailand

## Abstract

Cost-effective reduced graphene oxide sheets decorated with magnetite (Fe_3_O_4_) nanoparticles (Fe_3_O_4_-rGO) are successfully fabricated via a chemical vapor deposition (CVD) technique using iron (III) nitrate as an iron precursor, with glucose and CH_4_ as carbon sources, and NaCl as a supporting material. TEM analysis and Raman spectroscopy reveal hierarchical nanostructures of reduced graphene oxide (rGO) decorated with Fe_3_O_4_ nanoparticles. Fe *K*-edge x-ray absorption near edge structure (XANES) spectra confirm that the nanoparticles are Fe_3_O_4_ with a slight shift of the pre-edge peak position toward higher energy suggesting that the fabricated Fe_3_O_4_ nanoparticles have a higher average oxidation state than that of a standard Fe_3_O_4_ compound. The hierarchical Fe_3_O_4_-rGO is found to exhibit an excellent catalytic activity toward the reduction of triiodide to iodide in a dye-sensitized solar cell (DSSC) and can deliver a solar cell efficiency of 6.65%, which is superior to a Pt-based DSSC (6.37%).

## Introduction

Graphene is one of the most attractive two-dimensional nanostructured materials, which is extensively employed in a variety of applications due to its many exceptional properties such as high electrical conductivity, large specific surface area and high chemical and mechanical stabilities^1^. In a dye-sensitized solar cell (DSSC) application^2^, a material with high catalytic activity and conductivity is desirable for use as a counter electrode replacing expensive and scarce Pt. Graphene or graphene-related nanomaterials are ideally suited for this purpose in DSSCs because of their high electrical conductivity and large specific surface area.

Among the proposed Pt-free counter electrodes, carbonaceous materials^3–7^, conductive organic polymers^8^, metal sulfides^9–12^, metal nitrides^13,14^, metal carbides^15,16^ and metal oxides^17,18^. Fe oxides are one of the most abundant and environmentally-friendly substances whose well-known forms include magnetite (Fe_3_O_4_), hematite (α-Fe_2_O_3_) and maghemite (γ-Fe_2_O_3_). It is notable for its catalytic properties^19,20^. The superior catalytic activity of rust (α-Fe_2_O_3_) toward triiodide reduction was theoretically predicted and experimentally confirmed to be comparable to that of Pt^19^. Moreover, Fe_3_O_4_ and γ-Fe_2_O_3_ nanosheets were also noted for their superior electrocatalytic activities and photovoltaic performance in DSSCs compared to α-Fe_2_O_3_^20^. This is attributed to the presence of equal numbers of divalent and trivalent iron ions (Fe^2+^ and Fe^3+^) in octahedron sites in a cubic inverse spinel structure of Fe_3_O_4_. This provides for electron hopping transport giving rise to its lower resistance compared to other crystal phases (α-Fe_2_O_3_ and γ-Fe_2_O_3_)^21,22^. Therefore, the synergetic effects of high electrical conductivity and catalytic activity can be achieved from a composite of graphene and Fe_3_O_4_, enhancing the photovoltaic efficiency of DSSCs.

Many studies of graphene-based Fe_3_O_4_ nanocomposites have been previously reported. Most of them used reduced graphene oxide-Fe_3_O_4_ (rGO-Fe_3_O_4_) that exhibited outstanding electrochemical properties and were employed as supercapacitor electrode materials^23–25^. These imply the feasibility of the composites to promote electrocatalytic activities toward triiodide reduction in the case of DSSCs. Recently, highly dispersed Fe_3_O_4_ nanoparticles on reduced graphene oxide (rGO) have shown superior catalytic activity in reducing triiodide to iodide in DSSCs. This resulted in the highest ever reported efficiency of 9% (Pt ~ 9.46%)^26^.

rGO is a chemically derived graphene obtained from reducing graphene oxide (GO). It is considered a cost-effective graphene-based material that offers many advantages including good dispersibility in water enabling solution processability and versatile properties via specialized chemical functionalization^27^. Fe_3_O_4_-rGO composite materials were fabricated using various methods and typically a GO solution was employed as a precursor, which was generally prepared by a modified Hummers’ method^24,25,28–30^.

In this work, an excellent counter electrode material made of a Fe_3_O_4_-rGO nanocomposite was fabricated via a CVD based technique using glucose and CH_4_ as carbon sources and NaCl crystal as a supporting material. The as-synthesized Fe_3_O_4_-GO nanocomposites were achieved by dissolving the CVD product in water to remove NaCl crystals. It was then thermally reduced to form Fe_3_O_4_-rGO nanocomposites. Unlike previous fabrication methods of rGO composites, GO precursor is not required. The obtained Fe_3_O_4_-rGO exhibited excellent electrocatalytic activity and power conversion efficiency that was superior to the Pt DSSC. The fabrication and characterization of the Fe_3_O_4_-rGO nanocomposite are presented in this paper. The photovoltaic performance and electrochemical properties of the Fe_3_O_4_- rGO DSSC were investigated.

## Results and Discussion

### Fabrication of Fe_3_O_4_- rGO nanocomposite

**Figure 1:**
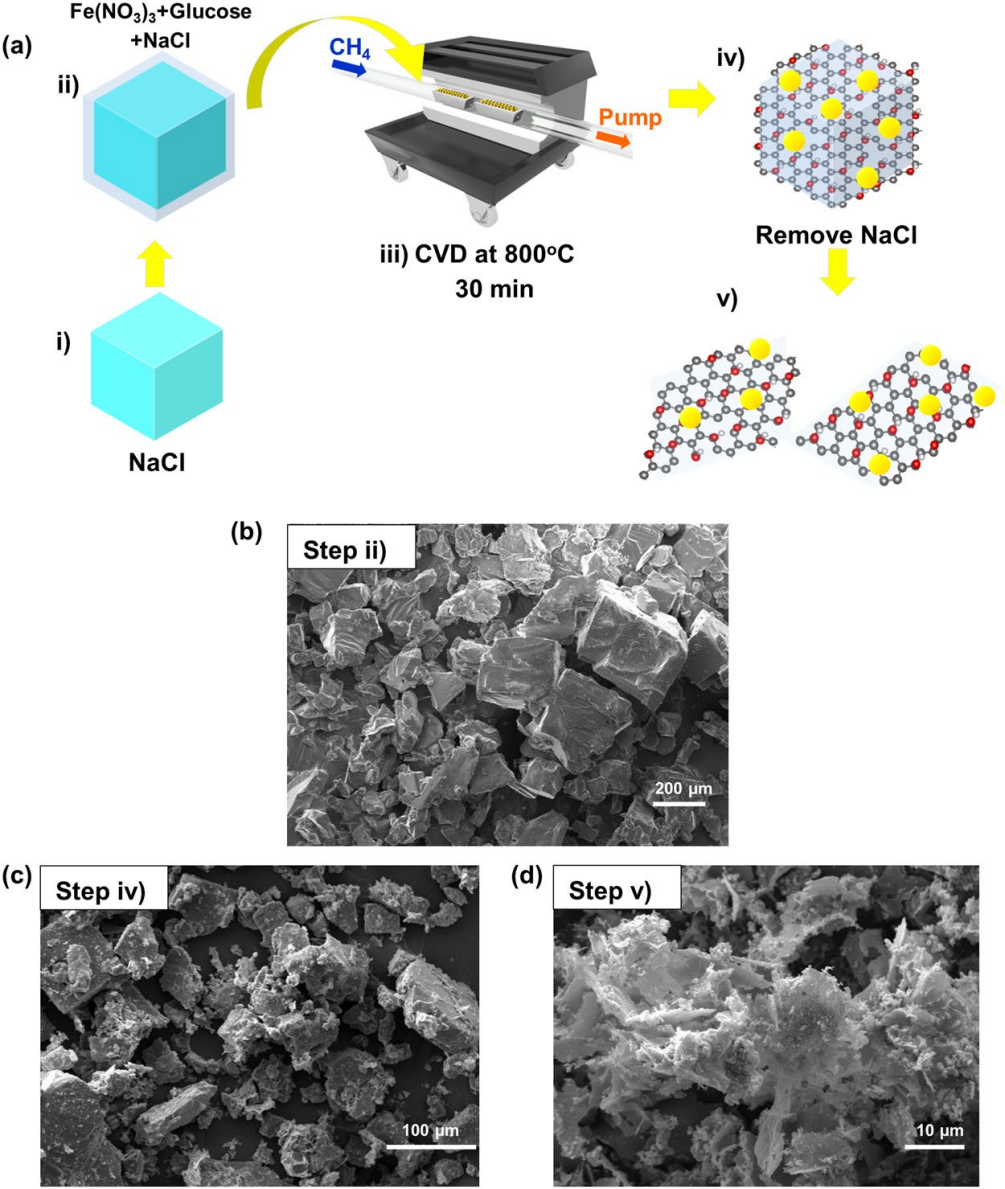
(**a**) Schematic diagram of synthesis process of the FGC sample, (**b**) SEM image of the precursor powder containing Fe(NO_3_)_3_, glucose and NaCl, (**c**) and (**d**) SEM images of as-synthesized CVD product before and after removing NaCl crystals.

The Fe_3_O_4_-rGO nanocomposite was synthesized by a CVD technique as described by the schematic diagram in Fig. 1(a). The CVD system consists of a tube furnace with a quartz tube of 2 cm diameter and 100 cm long, vacuum pump, and flow system for CH_4_ and Ar buffer gas. This CVD system was employed to prepare all the samples in this work. The precursors of the CVD reaction were prepared by dissolving Fe(NO_3_)_3_·9H_2_O (0.50 g), glucose (0.50 g) and NaCl (9 g) in 10 ml DI water. The mixture was dried in an oven at 80 °C for 24 h. Then the products were ground to obtain very fine powders that were used as precursors for the CVD process (step i). After placing an alumina combustion boat containing the precursor powders in a tube furnace, the system was purged with Ar for 30 min before the furnace was heated. Then the furnace temperature was raised from room temperature to 800 °C with a flow of Ar to maintain the constant pressure of 1 torr. When the temperature reached 800 °C, Ar gas was stopped. The furnace was then maintained at this reaction temperature with a flow of CH_4_ at constant flow rate of 2 sccm for 30 min (step ii). After the completion of reaction, the flow of CH_4_ was stopped and the furnace was cooled down to room temperature under the same Ar flow condition.

This sample was referred to as “FGC”, where F, G, and C represent Fe(NO_3_)_3_·9H_2_O, glucose and CH_4_, respectively.

Additionally, other composites were synthesized using the precursors prepared from the following mixtures. These include (*i*) glucose and NaCl with CH_4_ (without Fe(NO_3_)_3_·9H_2_O) referred to as “GC”, (*ii*) Fe(NO_3_)_3_·9H_2_O, glucose and NaCl (without CH_4_) called as “FG”, (*iii*) Fe(NO_3_)_3_·9H_2_O, NaCl and CH_4_ (without glucose) referred to as “FC”, and, (*iv*) Fe(NO_3_)_3_·9H_2_O and NaCl (without glucose and CH_4_) called as “F”. After the products were cooled to room temperature, they were washed with DI water for several times to remove NaCl (step iii). The products then were centrifuged and dried at 80 °C to obtain the nanocomposite powders (step iv).

### Nanostructural characterizations

The SEM images of the NaCl-Glucose-Fe precursor powders, the as-synthesized powder (CVD resultant powders) before and after removing NaCl are shown in Fig. 1(b–d), respectively.

**Figure 2:**
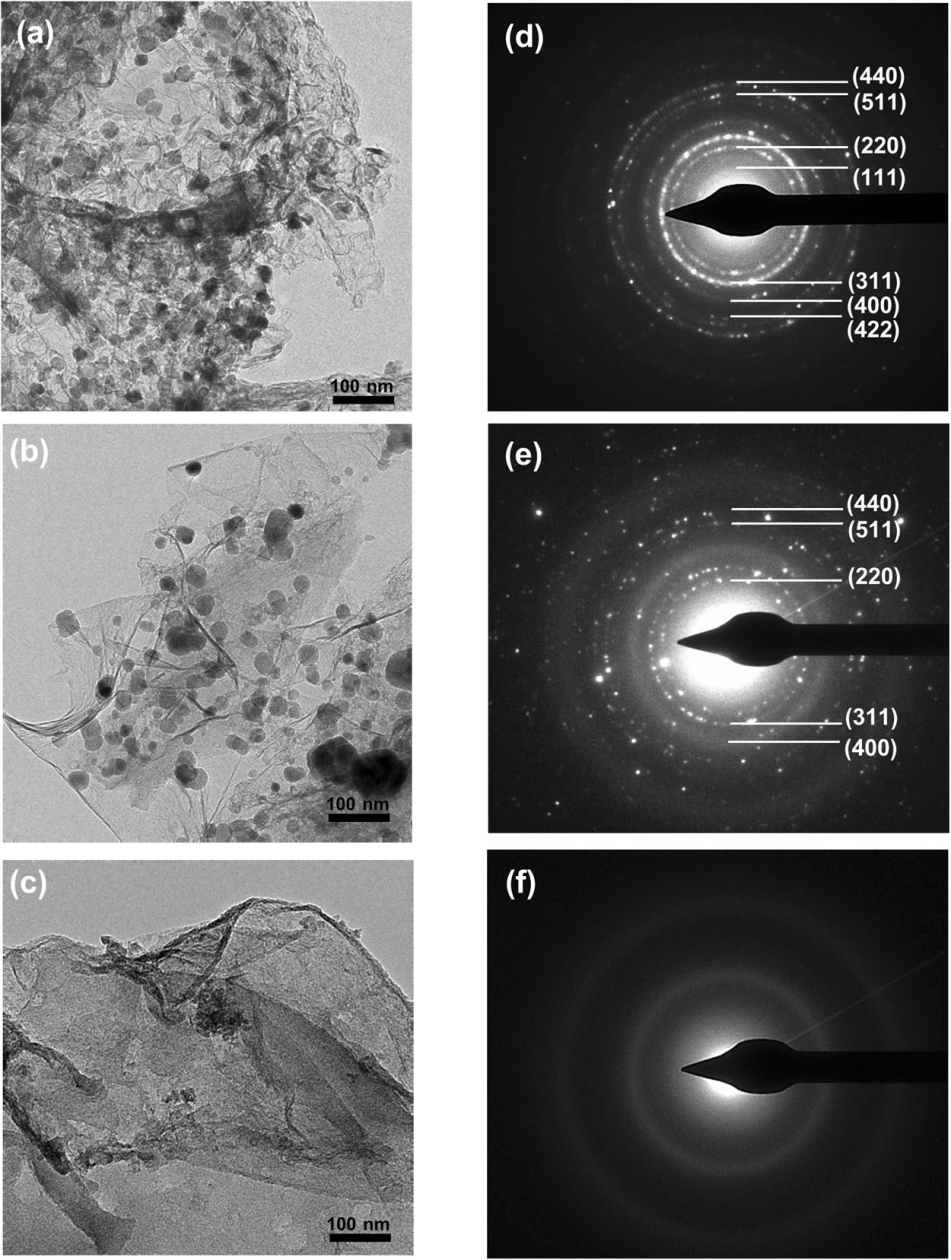
TEM images of the annealed (**a**) FGC, (**b**) FG and (**c**) GC samples and their SAED patterns (**d**); (**e**) and (**f**) respectively.

The TEM images of the annealed FGC, FG and GC samples are presented in Fig. 2. Sheet structures of carbon films decorated with nanoparticles were observed in FGC and FG samples, as shown in Fig. 2(a,b), respectively. Nanoparticles with sizes ranging from 10–30 nm in the FGC sample and those with a slightly larger size distribution (from 10-50 nm) in the FG sample were Fe_3_O_4_ as indexed from the SAED patterns in Fig. 2(d,e). The measured *d*-spacings from the patterns correspond to (111), (220), (311), (400), (422), and (511) planes of a cubic Fe_3_O_4_ (JCPDS file No. 19-0629). The observed sheet structures in FGC, FG and GC samples were amorphous carbon since there was no diffraction from a graphite structure detected in the SAED pattern (Fig. 2(d–f)). To confirm the crystal structure observed in TEM, XRD analysis of the FGC, FG and GC samples was carried out which showed a consistent result with the TEM as presented in Fig. S1 of the supplement information. The FGC features are distinct in the FG sample in that thin carbon films in the FGC sample had hierarchical structures, whereas those found in the FG sample were relatively flat. This difference in carbon film morphology can be ascribed to the use of CH_4_ for the CVD reaction in the FGC sample. In the case of the GC sample, which was prepared to investigate the effects of Fe in the composite, carbon films similar to those in the FG sample were observed as shown in Fig. 2(c). For the FC and F samples, Fe_3_O_4_ nanoparticles were detected with relatively large particle sizes of 50–80 nm, as shown in Fig. S2 of the supplementary information.

The role of NaCl crystals on the fabrication of the nanocomposites was also investigated by using the precursor powers (only glucose powders and a mixture of glucose and FeNO_3_) prepared without NaCl crystals for CVD reaction. It was found that without NaCl supporting material the nanocomposite was not deposited; the decomposed carbon molecules were carried away by pressure gradient. After several attempts, we failed to produce the sample without the presence of NaCl supporting material and hence the results of those samples were not included in this report.

**Figure 3:**
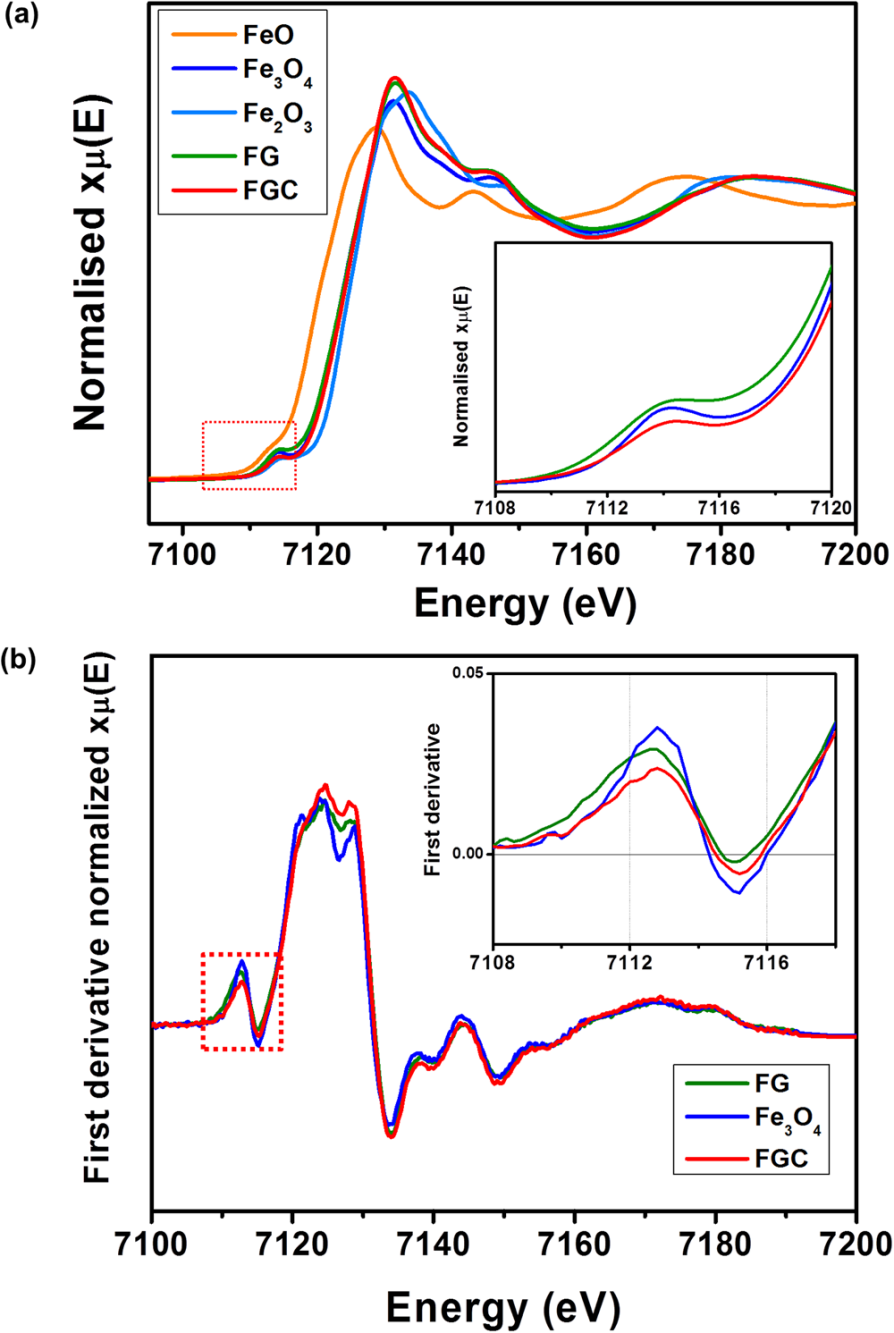
(**a**) Normalized and (**b**) first derivative Fe *K*-edge XANES of the annealed FGC and FG samples compared with standard samples of FeO, Fe_2_O_3_ and Fe_3_O_4_.

XANES spectroscopy is a powerful technique to investigate the local environment of the Fe atom. Fe *K*-edge XANES spectra of the FGC and FG samples, as well as the standard samples of FeO, Fe_2_O_3_, and Fe_3_O_4_ are shown in Fig. 3(a). The XANES spectra of the FGC and FG samples had very similar shapes to that of Fe_3_O_4_ standard. The pre-edge features of the *K*-edge of transition metal compounds have been found to be affected by the oxidation state and coordination environment of the atom of interest^31–33^, which can be determined from the pre-edge position and the height of the peak^34^. The pre-edge position can thus be used to probe the average Fe-redox state^31^. The pre-edge peak at ~7114 eV is related to the transition from 1*s* to 3*d*^33^. Its shift toward a higher energy indicates an increased oxidation state^34^. The pre-edge peak of the FGC sample was at a higher energy than that of the Fe_3_O_4_ standard, but lower than that of the FG sample, as shown in the first derivative intensity plot of Fig. 3(b). The shift toward a higher energy in the FG sample suggests a larger numbers of Fe^3+^ ions present in the sample than those of the FGC sample and the Fe_3_O_4_ standard. Fe_3_O_4_ has a cubic inverse spinel structure, where Fe^3+^ ions occupy tetrahedral sites and equal numbers of Fe^3+^ and Fe^2+^ ions occupy the octahedral sites. Its pre-edge peak intensity is approximately a weighted average of tetrahedral and octahedral intensities^35^. Since the nanoparticle size controls the local order, the pre-edge intensity can also be used to determine the crystallite size of the sample^32,34^. A lower pre-edge intensity indicates a smaller average particle size of the Fe_3_O_4_ in the FGC sample than those in the reference and the FG samples, respectively. This is consistent with the TEM results shown in Fig. 2.

**Figure 4:**
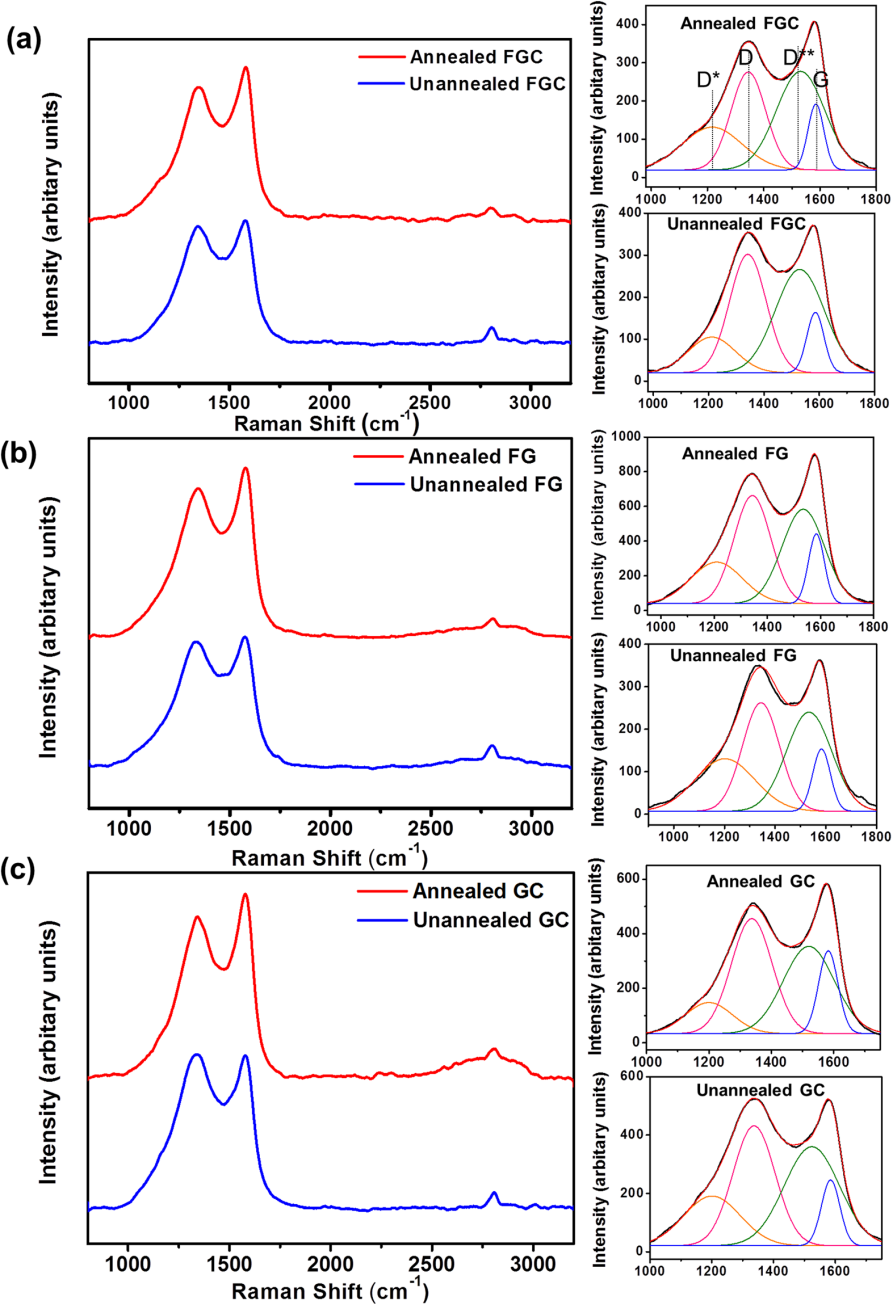
Raman spectra with the deconvolution of D and G peaks (right panels) of the unannealed and annealed (**a**) FGC, (**b**) FG and (**c**) GC samples.

Raman spectroscopy is an excellent technique for characterization of carbon nanomaterials. The Raman spectra in Fig. 4 are from the as-synthesized and as-annealed (at 480 °C for 1 h) FGC, FG and GC samples are denoted as “unannealed” and “annealed”, respectively (Fig. 4(a–c)). Two characteristic peaks of GO are visible in all samples at wavenumbers of ~1,340 and ~1,580 cm^−1^, which are defined as the D and G peaks, respectively. The D peak originates from out of plane vibrations due to the presence of structural disorders (structural defects, edge effects, and dangling sp^2^ carbon bonds) whereas the G peak arises from the in-plane vibrations of sp^2^ carbon atoms, which are common in graphitic materials^36^. The higher disorder in graphitic plane leads to a broader G band and a broad D band with higher relative intensity compared to that of the G band^37^. The broad D and G bands can be deconvoluted into four components: D*, D, D** and G centered at 1,200, 1,340, 1,530, and 1,585 cm^−1^, respectively. (The deconvoluted Raman spectra’s details are shown in Table S1 in the Supplementary Information.) D* and D** are, respectively, the sum and difference of carbon double bond stretching and hydrogen-carbon wagging modes, suggesting that the structures contained a reasonable number of defects^38,39^. Additionally, another signature band of graphitic sp^2^ material was observed at 2700–2800 cm^−1^_,_ called the 2D-band. The intensity ratio; I_D_/I_G_, can be used as a measure of defect density which can indicate the quality of GO^39^. The lower the I_D_/I_G_ ratio the higher quality of GO^40^. The rGO is therefore expected to have lower I_D_/I_G_ since the reduction process could remove the oxygen functional groups from GO and the repair of defects by recovery of hexagonal network^40^. Therefore, the electrical conductivity can be improved. The I_D_/I_G_ ratios of all the samples were found to decrease after annealing, i.e., from 1.96 to 1.48 in FGC, from 1.74 to 1.55 in FG and from 1.82 to 1.38 in GC samples. This suggests that upon annealing a considerable number of defects such as oxygen functional groups were removed from the GO sheets, which then became reduced GO (rGO)^37^.

Moreover, the sharper G peaks in all samples indicate the formation of sp^2^ hexagonal networks due to the self-recovery of carbon atoms^41^. It is notable that the 2D band became small modulated bumps that appeared in all the as-annealed samples, suggesting the samples formed wrinkles or corrugated structures which could be the consequence of the reduction process^38^. Post-annealing is, therefore, regarded as a crucial reduction method for removing defects and facilitating the restoration of sp^2^ carbon networks.

**Figure 5:**
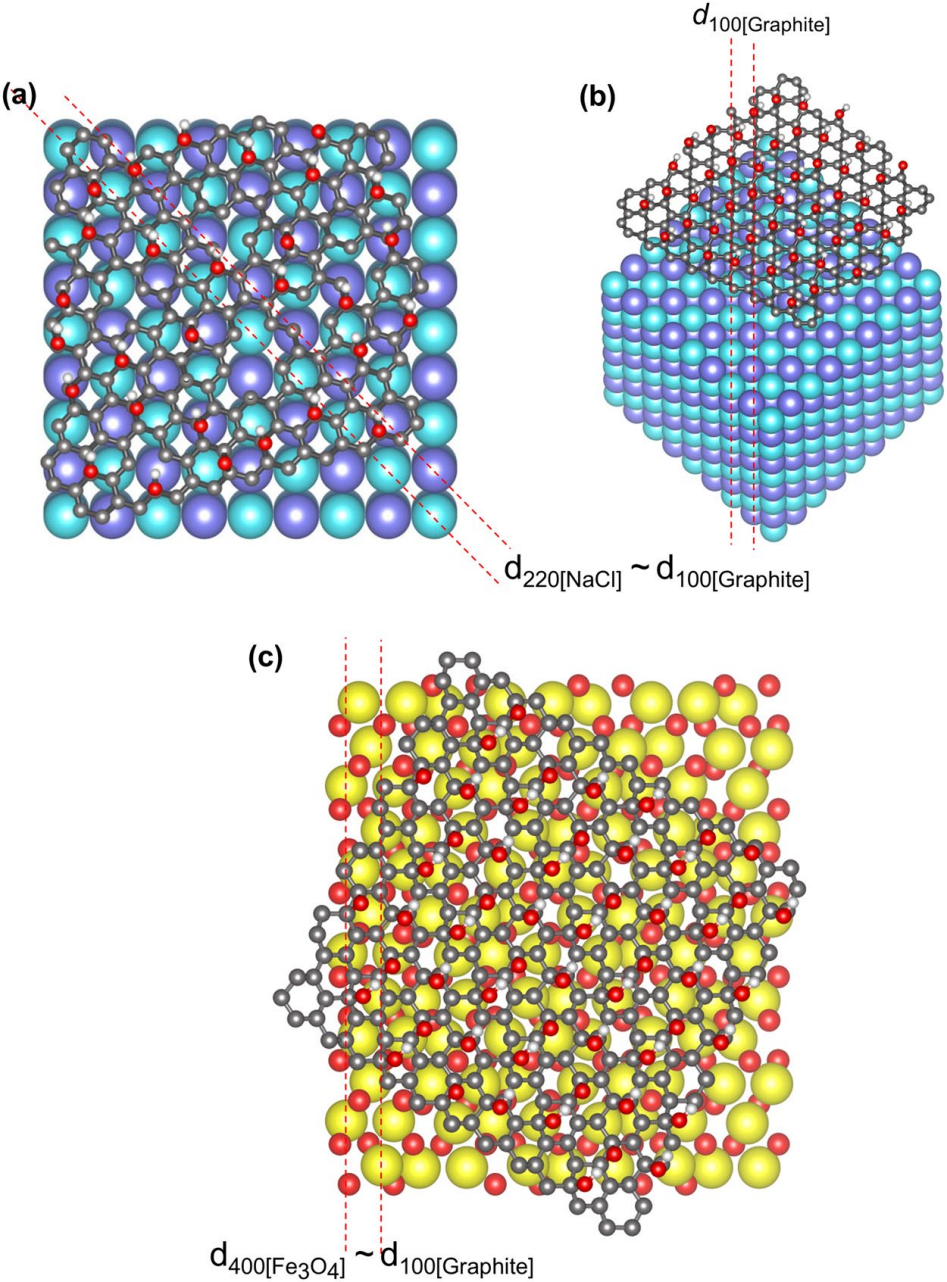
(**a**) and (**b**) Schematic of growth mechanism of GO on a NaCl crystal in top view and side view, respectively, and (**c**) GO on a Fe_3_O_4_ nanoparticle.

From these results, we propose a growth mechanism of the Fe_3_O_4_-rGO in the FGC sample as shown in the schematic presentation of Fig. 5. During the CVD process, glucose (C_6_H_12_O_6_) is primarily reduced (oxygen and hydrogen atoms are removed) and this carbon source would form a thin carbon layer covering the NaCl crystal surface. NaCl crystals act as seeding templates for carbon (reduced glucose) to transform it into a hexagonal GO network at an elevated temperature (800 °C). Simultaneously, nucleation of Fe_3_O_4_ on the GO network took place. The formation of GO on both NaCl and Fe_3_O_4_ is possible due to the *d*-spacing matches of the three materials as listed in Table 1. A hexagonal network of carbon atoms can form on the {100} planes of NaCl as the *d*-spacing matches between the (100) planes of graphite and the (220) planes of NaCl, as well as those of the (400) planes of Fe_3_O_4_. In the presence of CH_4_, another carbon source, the disassociated carbon atoms continue to grow from the GO sheet edges or nucleate on Fe_3_O_4_ nanoparticles generating a hierarchical-like structure as shown in Fig. 2(a). This was detected in the FGC sample but not in the GC sample. Thus, it can be deduced that Fe_3_O_4_ nanoparticles can act as another nucleation site for GO formation.

**Table 1:** *d*-spacings of NaCl, Graphite and Fe_3_O_4_.

NaCl		Graphite		Fe_3_O_4_	
*d*-spacing (Å)	Planes	*d*-spacing (Å)	Planes	*d*-spacing (Å)	Planes
3.26	(111)	3.38	(002)		
2.82	(200)			2.96	(220)
1.99	(220)	2.11 2.02	(100) (101)	2.10	(400)
1.63	(222)	1.69	(004)	1.71 1.61	(422) (511)
1.41	(400)			1.42	(531)
1.26	(420)	1.23	(110)	1.27	(622)
1.15	(422)	1.15	(105)	1.12	(642)

### DSSCs performance

**Figure 6:**
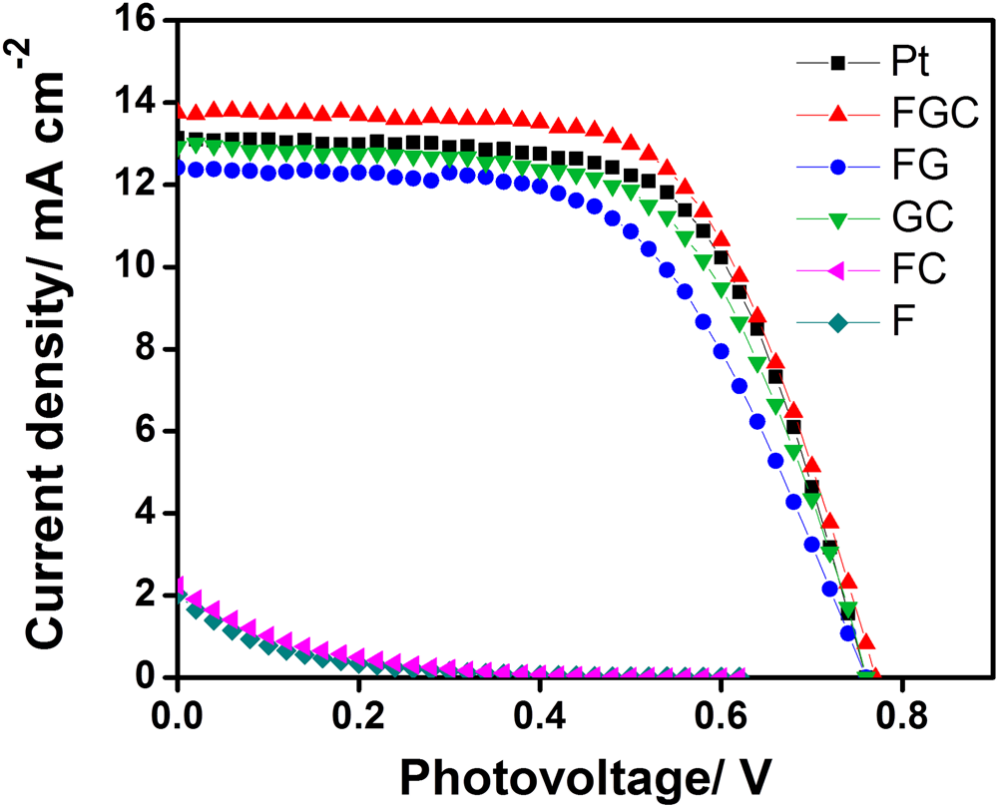
Photocurrent density-voltage (*J-V*) curves of the DSSCs with FGC, FG, GC, FC, and Pt counter electrodes.

The posted annealed FGC, FG, GC, FC, F electrodes and a Pt electrode were used as counter electrodes in dye-sensitized solar cell devices. The preparation of counter electrode, working electrode, DSSC assembly and cell characteristic measurements are described in the method section. The photocurrent density-voltage (*J-V*) curves and photovoltaic characteristics of the DSSCs are presented and summarized in Fig. 6 and Table 2, respectively. It was found that the DSSCs with an FGC CE exhibited excellent performance, superior to the Pt CE. The highest achieved power conversion efficiency (*η*) from FGC DSSCs was 6.65%, which surpassed that of Pt DSSCs (6.37%). The DSSCs with GC and FG CEs also showed promising solar cell efficiencies of 5.99% and 5.41%, respectively. Alternatively, the photovoltaic efficiencies were very low in the FC and F DSSCs. The maximum power conversion efficiency of the FGC DSSC can be deduced from the highest *J*_*sc*_ of 13.74 mA cm^−2^, which was greater than that of Pt DSSC (13.16 mA cm^−2^). The *V*_*oc*_ values of all DSSCs were comparable and in the range of 0.76–0.77 V.

**Table 2:** Summary of open-circuit voltage (*V*_*oc*_), short-circuit current density (*J*_*sc*_), fill factor (FF) and solar cell efficiency (*η*) of FGC (heirarchical Fe_3_O_4_-rGO), GC (rGO), FG (Fe_3_O_4_-rGO), FC, F and Pt DSSCs, and the parameters derived from CV and EIS spectra of FGC, FG, GC and Pt CEs including E_pp_, *R*_*s*_, and *R*_*ct*_.

Electrode	*J*_*sc*_(mA cm^−2^)	V_oc_ (V)	FF	*η* (%)	E_pp_ (mV)	*R*_*s*_ *(Ω)*	*R*_*ct*_ *(Ω)*
FGC	13.74	0.77	0.63	6.65	324	7.22	5.3
GC	12.80	0.76	0.61	5.99	680	8.50	6.5
FG	12.38	0.76	0.58	5.41	274	9.45	24
FC	1.99	0.67	0.08	0.10	—	—	—
F	2.03	0.64	0.07	0.07	—	—	—
Pt	13.16	0.76	0.64	6.37	453	7.23	5.4

The highest achieved photovoltaic performance was from the hierarchical rGO with Fe_3_O_4_ nanoparticles in the FGC sample. In order to investigate the role of each component in the FGC nanocomposite, the correlation of solar cell efficiencies and microstructural characteristics of the three counter electrode materials including FGC, GC and FG samples are considered. In this respect, it is found that the rGO in the GC sample gave the efficiency of upto 5.99%, thus it can be deduced that rGO contribute mainly to the DSSC performance, since rGO is one of components in the FGC CE. The relatively high performance of the produced rGO counter electrode is due to its high electrical conductivity and high specific surface area which are good for electrocatalytic activity toward the reduction of triiodide. Our result showed a consistent trend with many previous studies on using graphene and graphene-related materials as counter electrode^7,42–48^.

However, in the rGO with Fe_3_O_4_ in the FG sample, the solar cell efficiency dropped to 5.41%. Please note that for the counter electrode preparation the amount of counter electrode materials were controlled by weight. Therefore, GC counter electrode would contain higher rGO content than the FG one that contained rGO plus Fe_3_O_4_ nanoparticles, and hence giving rise to the higher *J*_*sc*_ and solar cell efficiency.

Please also note that rGO sheets both in the GC and FG samples were not in hierarchical structure like those in the FGC sample. Therefore, the surplus efficiency of the FGC counter electrode is therefore attributed to the increased specific surface area of hierarchical rGO and the enhanced redox activity by Fe_3_O_4_ catalyst.

**Figure 7:**
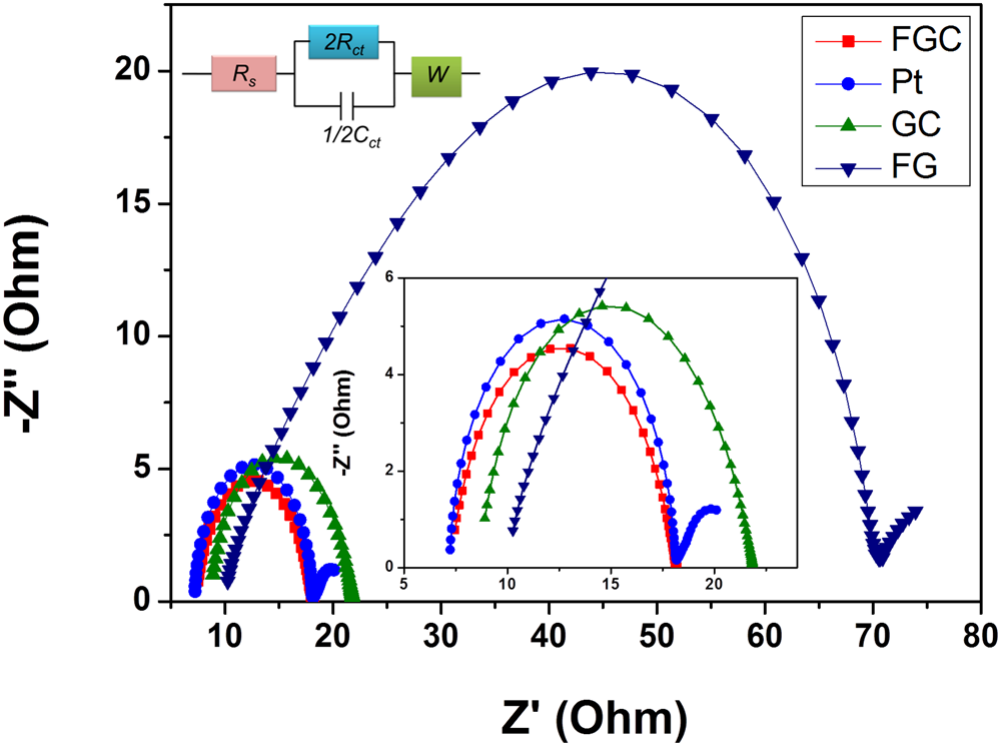
Nyquist plots of symmetrical cells based on the FGC, FG, GC, and Pt counter electrodes.

EIS measurement was also carried out to investigate electrochemical properties of the FGC, GC and FG CEs using symmetrical cells (CE/electrolyte/CE). The Nyquist plots of the fabricated CEs and Pt with their equivalent circuit are shown in Fig. 7. It sees that the FGC, FG and Pt CEs exhibit single semicircle curve. The series resistance (*R*_*s*_) correlates with FTO resistance and the contact resistance of CE material and FTO surface, which can be determined from the intercept on the real axis. The *R*_*s*_ values of the FGC, FG, GC and Pt CEs were 7.22, 9.45, 8.50 and 7.23 Ω, respectively. The resistance-capacitance (*RC*) network of the electrode/electrolyte interface includes the charge-transfer resistance (*R*_*ct*_) and the corresponding capacitance (*C*_*ct*_). The lower *R*_*ct*_ value the better charge-transfer between CE and electrolyte, and hence the more effective catalytic activity for triiodide reduction. Among the three CE materials, the FGC electrode had the lowest *R*_*ct*_ of 5.3 Ω, lower than that of Pt CE which was 5.4 Ω, and lower than those of GC and FG CEs (~6.5 and 24 Ω, respectively). The *R*_*s*_ and *R*_*ct*_ derived from EIS data are summarized in Table 2 and the fitted EIS curves are presented in Fig. S3 of the supplementary information.

**Figure 8:**
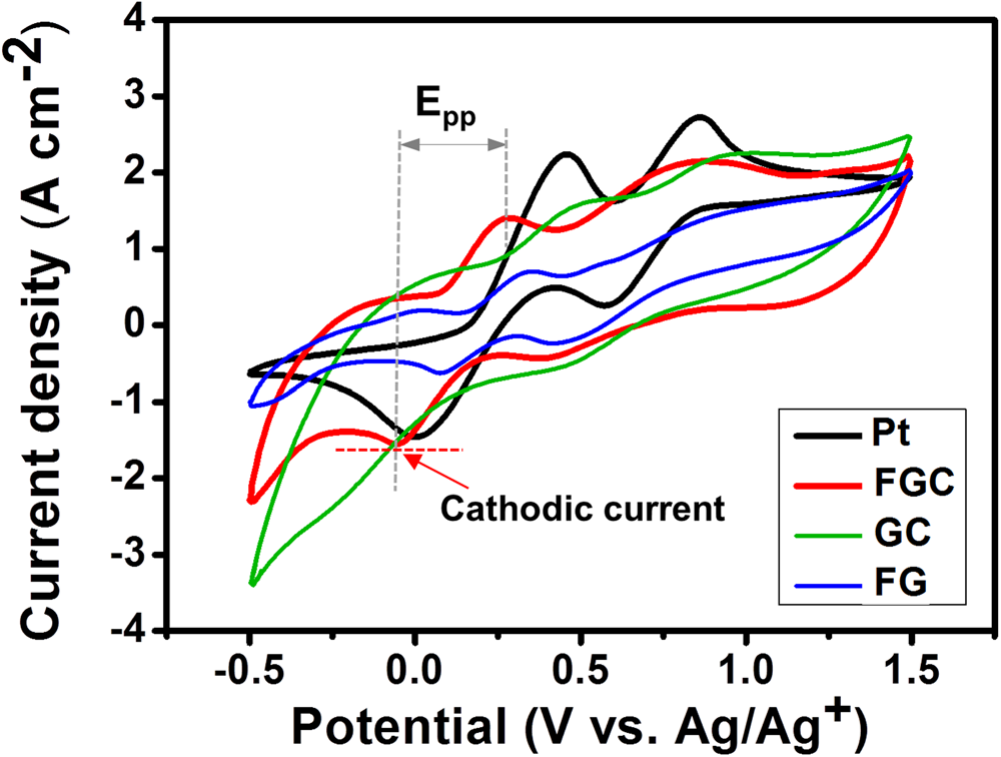
Cyclic voltammograms of the FGC, FG, GC and Pt counter electrodes.

The catalytic activities of the CE materials toward the triiodide reduction in DSSC were investigated by cyclic voltammetry (CV) to understand the variation in cell performance. The CV measurements were performed at a scan rate of 20 mV s^−1^ using the FGC, GC, FG and Pt samples as working electrodes. The results are shown in Fig. 8. For the Pt CE, two pairs of redox peaks were typically visible in the CV. The left and the right pairs corresponded to the redox reactions represented in Equations (1) and (2), respectively.

1 $${I}_{3}^{-}+2{e}^{-}\leftrightarrows 3{I}^{-}$$

2 $$3{I}_{2}+2{e}^{-}\leftrightarrows 2{I}_{3}^{-}$$

The role of a CE is to catalyze the reduction of ${I}_{3}^{-}$ to ${I}^{-}$, and this corresponds to the left reduction peak. The low value of the peak to peak separation (E_pp_), which is inversely correlated with the standard electrochemical rate constant of the redox reaction, and high cathodic peak current density are required for the excellent electrocatalytic performance of a CE. The GC CE had the highest cathodic peak current with a large E_pp_ value of 680 mV, whereas the FGC and FG CEs had smaller E_pp_ values of 324 and 274 mV, respectively. The E_pp_ values of FGC and FG CEs were smaller than that of Pt CE which was 453 mV, indicating that the Fe_3_O_4_ can provide high redox reaction rate.

The role of Fe_3_O_4_ nanoparticles in counter electrode is to enhance the kinetic of the triiodide reduction process in electrolyte solution by the redox reaction in Equation (3) below.

3 $$F{e}^{3+}+{e}^{-}\leftrightarrow F{e}^{2+}$$

Fe_3_O_4_ contain unequal amount of Fe^3+^ and Fe^2+^ ions in a unit cell. Fe^3+^ ion is an oxidizing agent or an electron acceptor. Electrons from rGO or FTO can be captured by Fe^3+^ ions which are then reduced to Fe^2+^ ions. Fe^2+^ ions act as electron donor or a reducing agent for triiodide (*I*_3_^−^) to become *I*^*−*^ by the reaction in Equation (4) below^49^.

4 $$2F{e}^{2+}+{I}_{3}^{-}\leftrightarrow 2F{e}^{3+}+3{I}^{-}$$

According to the XANES results, the high oxidation state of FG CE implies a higher Fe^3+^ to Fe^2+^ ratio. The larger number of electron acceptors, the faster electron can be transferred, as seen by its corresponding lowest E_pp_ value. The correlation of E_pp_ and XANES results suggests that Fe_3_O_4_ nanoparticles play a crucial role in enhancing kinetic of the triiodode reduction by providing fast electron transfer upon Fe^3+^/Fe^2+^ redox process.

Please note that the rGO in the FG sample was relatively flat compared to that of the FGC sample which had hierarchical structure, which had more edge sites than that of the flat rGO in FG CE. It has been reported that the edge sites of carbon films are more electrocatalytically reactive than the basal plane^50^. Consequently, despite the slightly higher E_pp_ value than the FG, the high specific surface area, good electrical conductivity and more electrochemically active edge sites of hierarchical rGO in the FGC sample account for its high cathodic current and low *R*_*ct*_ and hence giving rise to the excellent counter electrode performance.

## Conclusion

The hierarchical structures of rGO decorated with Fe_3_O_4_ nanoparticles or Fe_3_O_4_–rGO nanocomposites with remarkably high catalytic performance toward triiodide reduction were successfully synthesized. This was done via a CVD based fabrication technique using glucose and CH_4_ as carbon sources and NaCl crystals as a supporting material. A hierarchical structure of Fe_3_O_4_–rGO (FGC sample) was not observed when glucose was used as the only carbon source (FG sample). Instead, relatively flat surfaced Fe_3_O_4_-rGO nanosheets were obtained. The Fe_3_O_4_–rGO CE with hierarchical nanostructures was found to exhibit a maximum energy conversion efficiency of 6.65%, which was superior to a Pt-based DSSC (6.37%). This outstanding performance is attributed to a combination of two factors. First is its superior electrocatalytic activity toward triiodide reduction due to the presence of Fe^3+^/Fe^2+^ from Fe_3_O_4_ nanoparticles. The second is good electrical conductivity due to a high charge transfer rate with a large number of edge sites and correspondingly higher surface area of the hierarchical rGO nanostructure. This achievement could create opportunities for development of highly efficient DSSC devices with low production costs.

## Methods

### Counter electrode preparation

The counter electrodes for the DSSCs were prepared by drop coating a 250 µl suspension of the fabricated nanocomposite in acetone (1 mg/ml) on fluoride doped tin oxide (FTO, 7 Ω/square) glass pieces forming four consecutive layers. Then, these films were dried at 80 °C for 1 hour and heated in Ar at 480 °C for 1 hour to reduce the GO and improve the adhesion of the CE material to the FTO surface.

### Working electrode preparation

The TiO_2_ anode was prepared using a previously reported screen printing method^17^. Briefly, the TiO_2_ films were fabricated using commercial TiO_2_ pastes, PST-18NR and PST-400C (JGC Catalysts and Chemicals Company, Japan) on FTO substrates. Then, the FTO/TiO_2_ samples were annealed at 500 °C for 1 h, and treated with UV radiation for 10 min. Next, the working electrodes were immersed in a dye solution consisting of 0.3 mM *cis-*bis-(isothiocyanato) bis (2, 2-bipyridyl-4, 4-dicarboxylato)-ruthenium(II)-bis-tetrabutylammonium (N-719, Solaronix), for 24 h to achieve a dye-sensitized electrode.

### DSSC assembly

Semi-closed DSSCs were assembled using TiO_2_ coated dye-sensitizer films as the working electrode (WE), and the prepared counter electrodes (CE) including those made from FGC, GC, FG, FC, F and Pt. Parafilm was used as a spacer between the working and counter electrodes. A I^−^/I_3_^−^ liquid electrolyte (0.05 M I_2_, 0.10 M LiI, 0.60 M of 1, 2-dimentyl-3-propylimidasolium iodide (MPI), 0.0025 M Li_2_CO_3_ and 0.50 M of 4-tert-butylpyridine (TBP) in acetonitrile) was injected into the cells.

### Film and cell characteristics

The film morphologies and crystal structures were characterized using scanning electron microscopy (SEM, LEO 1450 VP, Germany), transmission electron microscopy (TEM, FEI, TECNAI G^2^, the Netherlands), respectively. Raman spectroscopy was employed to investigate the carbon nanostructures using a triple-monochromator JOBIN YVON HORIBA T64000 spectrometer with a 532 nm laser excitation line. X-ray absorption near-edge structure (XANES) spectra for FGC and FC samples were acquired in transmission mode at beamline 1.1 W of the Synchrotron Light Research Institute (SLRI), Nakhon Ratchasima, Thailand. The photovoltaic performance of the DSSCs was measured using a solar simulator (PEC-L11, Japan) under a light intensity of 100 mW cm^−2^. Their electrocatalytic activity was analyzed using a cyclic voltammogram (CV, CS150 Electrochemical Workstation, Wuhan Corrtest Instrument Co., Ltd) with a three-electrode system, i.e., an Ag/AgCl electrode as the reference electrode, Pt film as the counter electrode and the fabricated materials (FGC, GC and FG) as the working electrodes at a scan rate of 20 mV s^−1^ in 10 mM LiI, 1 mM I_2_, and 0.1 M LiClO_4_ in an acetonitrile solution.

## Supplementary information

Supplementary Information for Publication
