# Supplementary material for: Hierarchical Fe3O4-reduced graphene oxide nanocomposite grown on NaCl crystals for triiodide reduction in dye-sensitized solar cells
Source: Sci Rep. 2019 Feb 6;9:1494. doi: 10.1038/s41598-018-38050-z (PMC6365545; doi:10.1038/s41598-018-38050-z)
Supplement: Supplementary file 1 — Supplementary Information for Publication [file 41598_2018_38050_MOESM1_ESM.docx]

**Supplementary information**

**Hierarchical Fe_3_O_4_-reduced graphene oxide nanocomposite grown on NaCl crystals for triiodide reduction in dye-sensitized solar cells**

Viyada Harnchana^1,2,3,4,^*, Sujinda Chaiyachad^1^, Samuk Pimanpang^2,3,4,5^, Chatree Saiyasombat^6^, Pornjuk Srepusharawoot^1,2,3,4^, and Vittaya Amornkitbamrung^1,2,3,4^

^1^ Department of Physics, Faculty of Science, Khon Kaen University, Khon Kaen 40002, Thailand

^2^ Institute of Nanomaterials Research and Innovation for Energy (IN-RIE), NANOTEC -KKU RNN on Nanomaterials Research and Innovation for Energy, Khon Kaen University, Khon Kaen, 40002, Thailand

^3^Intergrated Nanotechonology, Khon Kaen University, Khon Kaen, 40002, Thailand

^4^Thailand Center of Excellence in Physics, Commission on Higher Education, Bangkok 10400, Thailand

^5^ Department of Physics, Faculty of Science, Srinakharinwirot University, Bangkok 10110, Thailand

^6^ Synchrotron Light Research Institute (Public Organization), Nakhon Ratchasima 30000, Thailand

*Corresponding author. Tel: +6680 368 2040. E-mail: viyada@kku.ac.th

**Figure S1**. XRD patterns of the FGC, FG, GC samples with stick patterns of Fe_3_O_4_, Graphite and NaCl JCPDS references no. 190629, 010646 and 050628, respectively.

*** Fe_3_O_4_**

*** Graphite**

*** NaCl**

*****

*****

*****

*****

*****

*****

*****

*****

*****

*****

*****

*****

*****

*****

*****

FGC

FG

GC& residual NaCl

GC

**2θ (*deg*)**

**Intensity (arbiatary units)**

The XRD patterns of the annealed FGC, FG, and GC samples are present in figure R2-1. In the FGC and FG samples, XRD patterns consist of the diffraction peaks from (220), (311), (222), (400),(422), (511) and (440) planes of Fe_3_O_4_ at 2θ values of 30.2, 35.6, 37.2, 43.2, 53.6, 57.2 and 62.9^o^ respectively. The noisy background and lower peak intensities in the FGC sample than those of the FG could be attributed to the high amorphous carbon background from hierarchical rGO, which could also superimpose on the low intensity Fe_3_O_4_ peaks due to of the small crystalline size in the FGC sample. The similar results hav also been reported by some previous studies[^1^](#_ENREF_1)^,^[^2^](#_ENREF_2). The XRD pattern of the GC sample suggests amorphous structure, no diffraction peak from graphite is observed. However, in some GC samples the residual NaCl was detected which can be removed after washed with DI water for several times.

**References**

1 Silva, V. A. J. *et al.* Synthesis and characterization of Fe3O4 nanoparticles coated with fucan polysaccharides. *Journal of Magnetism and Magnetic Materials* **343**, 138-143, doi:https://doi.org/10.1016/j.jmmm.2013.04.062 (2013).

2 Hong, R. Y. *et al.* Synthesis, characterization and MRI application of dextran-coated Fe3O4 magnetic nanoparticles. *Biochemical Engineering Journal* **42**, 290-300, doi:https://doi.org/10.1016/j.bej.2008.07.009 (2008).


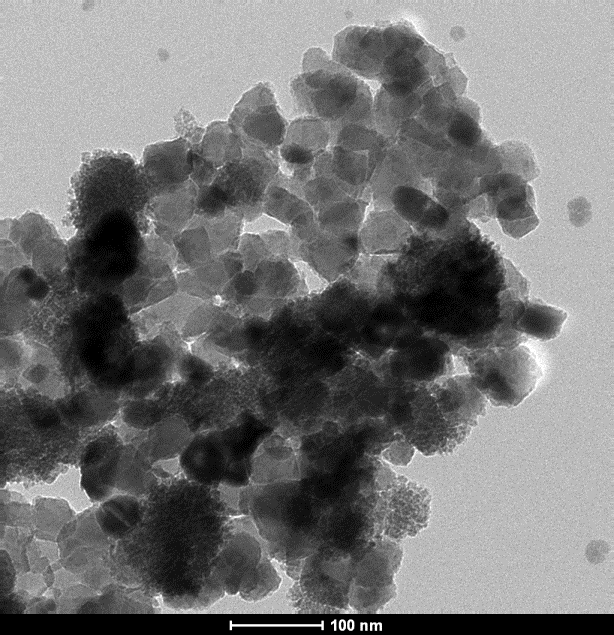

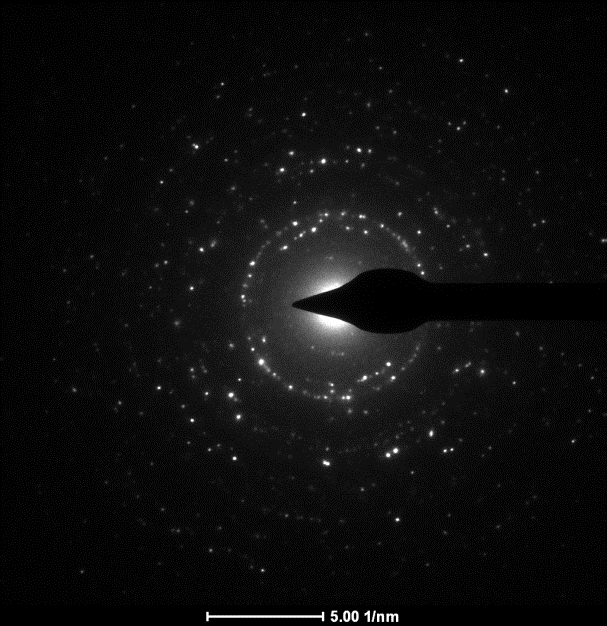

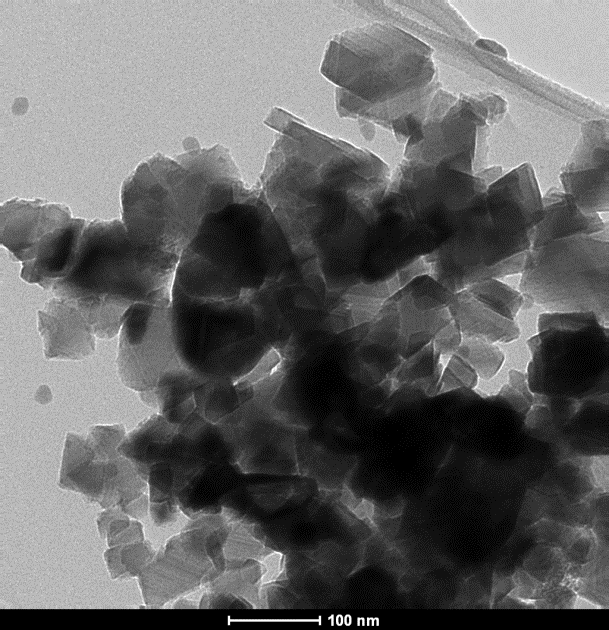

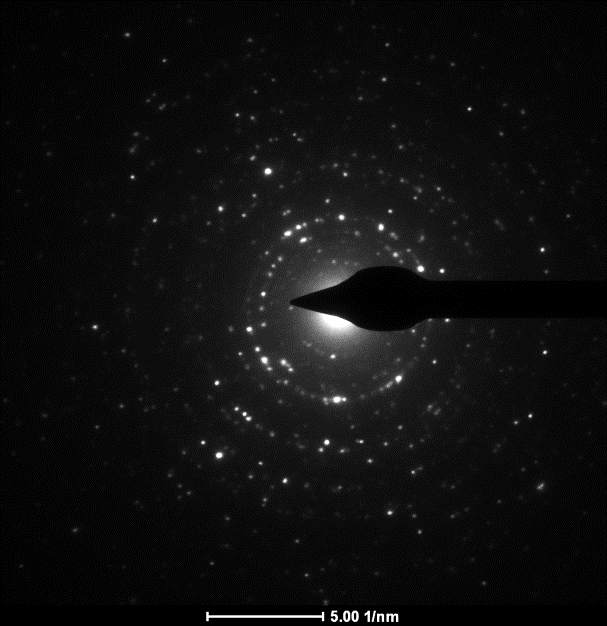


(a)

(b)

(c)

(d)

**Figure S**2. TEM images of (a) the FC, (b) F samples and their SAED patterns (c) and (d) respectively.

**Figure S**3. EIS spectra with fitted curves of the FGC, FG, GC, and Pt CEs.

| **Sample** | **Peak** | **Peak position** | **Area Fit** | **Area Fit %** | **Intensity** | **FWHM** | **I/I_G_** |
| --- | --- | --- | --- | --- | --- | --- | --- |
| FGC unannealed | D* | 1211.25 | 18169.39 | 13.87 | 85.28 | 200.15 | 0.59 |
|  | D | 1341.70 | 46843.64 | 35.77 | 282.33 | 155.87 | 1.96 |
|  | D** | 1529.34 | 54762.28 | 41.82 | 246.45 | 208.75 | 1.72 |
|  | G | 1585.76 | 11181.88 | 8.539 | 143.89 | 73.00 | 1 |
| FGC annealed | D* | 1216.17 | 29893.84 | 21.75 | 112.05 | 250.63 | 0.64 |
|  | D | 1344.94 | 39666.67 | 28.86 | 256.23 | 145.43 | 1.48 |
|  | D** | 1530.72 | 55649.86 | 40.49 | 257.78 | 202.81 | 1.49 |
|  | G | 1585.20 | 12236.05 | 8.90 | 172.39 | 66.68 | 1 |
| FG unannealed | D* | 1202.08 | 36264.38 | 24.51 | 123.65 | 275.53 | 0.84 |
|  | D | 1344.09 | 46634.39 | 31.53 | 255.44 | 171.51 | 1.74 |
|  | D** | 1533.27 | 52245.63 | 35.33 | 233.35 | 210.34 | 1.59 |
|  | G | 1582.73 | 12762.94 | 8.63 | 146.38 | 81.91 | 1 |
| FG annealed | D* | 1209.54 | 58591.07 | 19.15 | 239.80 | 229.52 | 0.59 |
|  | D | 1343.72 | 108294.27 | 35.40 | 623.03 | 163.29 | 1.55 |
|  | D** | 1534.94 | 108765.20 | 35.55 | 544.27 | 187.73 | 1.35 |
|  | G | 1584.29 | 30228.37 | 9.88 | 401.96 | 70.65 | 1 |
| GC unannealed | D* | 1200.71 | 39149.47 | 19.33 | 170.56 | 215.63 | 0.76 |
|  | D | 1336.61 | 70230.60 | 34.67 | 411.25 | 160.43 | 1.82 |
|  | D** | 1523.92 | 76243.47 | 37.64 | 339.49 | 210.98 | 1.50 |
|  | G | 1584.84 | 16921.59 | 8.35 | 225.39 | 70.528 | 1 |
| GC annealed | D* | 1200.04 | 21521.99 | 11.92 | 114.04 | 177.29 | 0.37 |
|  | D | 1337.46 | 69441.26 | 38.47 | 421.53 | 154.76 | 1.38 |
|  | D** | 1520.26 | 65817.99 | 36.46 | 320.01 | 193.22 | 1.05 |
|  | G | 1582.73 | 23710.09 | 13.14 | 303.57 | 73.37 | 1 |

**Table S1** the deconvolution data extracted from the Raman spectra in Fig. 4 including Peak position, area fit, peak intensity, FWHM and the intensity ratio I/I_G_.
